# Tuberous sclerosis complex: a complex case

**DOI:** 10.1101/mcs.a006182

**Published:** 2022-04

**Authors:** Ryan M. Powell, Sharon Pattison, Jiri C. Moravec, Basharat Bhat, Nada Guirguis, David Markie, Greg T. Jones, Jason Copedo, Cristin G. Print, Ian M. Morison, Alex Gavryushkin, Bronwyn Gray, Lisa J. Wyeth, Mike R. Eccles, Erin C. Macaulay

**Affiliations:** 1 Department of Pathology, University of Otago, Dunedin 9016, New Zealand;; 2 Department of Medicine, University of Otago, Dunedin 9016, New Zealand;; 3 Department of Computer Sciences, University of Otago, Dunedin 9016, New Zealand;; 4 Department of Surgical Sciences, University of Otago, Dunedin School of Medicine, Dunedin 9016, New Zealand;; 5 Grafton Clinical Genomics, University of Auckland, Auckland 1023, New Zealand;; 6 Department of Molecular Medicine and Pathology, University of Auckland, Auckland 1023, New Zealand;; 7 School of Mathematics and Statistics, University of Canterbury, Canterbury 8140, New Zealand;; 8 Tuberous Sclerosis Complex—New Zealand, New Zealand;; 9 The New Zealand LAM Charitable Trust, New Zealand

**Keywords:** duodenal carcinoma, pulmonary lymphangiomyomatosis, renal angiomyolipoma

## Abstract

Tuberous sclerosis complex (TSC) is an inheritable disorder characterized by the formation of benign yet disorganized tumors in multiple organ systems. Germline mutations in the *TSC1* (hamartin) or more frequently *TSC2* (tuberin) genes are causative for TSC. The malignant manifestations of TSC, pulmonary lymphangioleiomyomatosis (LAM) and renal angiomyolipoma (AML), may also occur as independent sporadic perivascular epithelial cell tumor (PEComa) characterized by somatic *TSC2* mutations. Thus, discerning TSC from the copresentation of sporadic LAM and sporadic AML may be obscured in TSC patients lacking additional features. In this report, we present a case study on a single patient initially reported to have sporadic LAM and a mucinous duodenal adenocarcinoma deficient in DNA mismatch repair proteins. Moreover, the patient had a history of Wilms’ tumor, which was reclassified as AML following the LAM diagnosis. Therefore, we investigated the origins and relatedness of these tumors. Using germline whole-genome sequencing, we identified a premature truncation in one of the patient's *TSC2* alleles. Using immunohistochemistry, loss of tuberin expression was observed in AML and LAM tissue. However, no evidence of a somatic loss of heterozygosity or DNA methylation epimutations was observed at the *TSC2* locus, suggesting alternate mechanisms may contribute to loss of the tumor suppressor protein. In the mucinous duodenal adenocarcinoma, no causative mutations were found in the DNA mismatch repair genes *MLH1, MSH2, MSH6*, or *PMS2*. Rather, clonal deconvolution analyses were used to identify mutations contributing to pathogenesis. This report highlights both the utility of using multiple sequencing techniques and the complexity of interpreting the data in a clinical context.

## INTRODUCTION

Tuberous sclerosis complex (TSC) is an autosomal inherited disorder characterized by the formation of hamartomas in almost all organ systems (Crino et al. 2006). Frequently benign in nature, hamartomas are composed of disorganized cells but otherwise typical for the organs in which they develop (Li et al. 2011). In addition to hamartomas, neurological abnormalities are typical. Indeed, the name tuberous sclerosis complex is derived from the presence of the characteristic cortical tubers, hypomyelinated hamartomas prominent in the cerebral folds (Pascual-Castroviejo et al. 2013). Further presentations of the disease vary between cases, with common manifestations including skin lesions, cardiac rhabdomyomas, renal angiomyolipomas (TSC-AML), and pulmonary lymphangioleiomyomatosis (TSC-LAM) (Crino et al. 2006; Gupta and Henske 2018). During the late 1980s and early 1990s, genetic linkage analyses identified variants in the *TSC1* (hamartin) and *TSC2* (tuberin) as causal for TSC (Fryer et al. 1987; Kandt et al. 1992). Subsequent somatic mutations in the remaining functional allele are postulated to drive tumorigenesis. Thus, *TSC1* and *TSC2* encode prototypical tumor-suppressor proteins (Kobayashi et al. 1997).

Like TSC, the *TSC2* locus frequently undergoes somatic loss-of-heterozygosity (LOH) mutations in perivascular epithelial cell tumors (PEComas) (Pan et al. 2006, 2008). PEComas encompass a diverse group of tumors united by markers of dual melanocytic and smooth-muscle cell differentiation pathways, including sporadic lymphangioleiomyomatosis (S-LAM) and sporadic angiomyolipoma (S-AML) (Martignoni et al. 2008). These smooth-muscle cells undertake a spindle morphology, and express alpha-smooth muscle actin (α-SMA) (Folpe and Kwiatkowski 2010). Meanwhile, melanocytic markers including HMB-45 and Melan-A are observed predominantly in cells with an epithelioid morphology (Folpe and Kwiatkowski 2010). Additional markers of adipose differentiation are commonly observed in AML (Lane et al. 2008). Interestingly, S-LAM is observed almost exclusively in women (Hohman et al. 2008; Ryu et al. 2012). Further similarities between LAM and AML include metastatic potential, with AML commonly observed in the hepatic system (Fricke et al. 2004). Although rare, duodenal manifestations of AML or other nondefined PEComas have been reported in the literature (Narayanaswamy et al. 2008; Wang et al. 2017). Therefore, distinguishing the copresentation of multiple sporadic PEComas including S-LAM and S-AML from the TSC-associated manifestations may be obscured in TSC patients lacking additional features (Northrup et al. 2013).

## RESULTS

### Clinical Presentation

Herein, we describe a patient discovered as part of a research cohort investigating the tissue of origin for S-LAM.

The patient initially presented at 13 yr of age with a painful and severely swollen abdomen, and subsequently underwent a nephrectomy, with the resected mass diagnosed as a Wilms’ tumor. This was followed by 5 wk of radiation and 15 mo of chemotherapy. The patient presented 3 yr later with bilateral pneumothoraces. At age 27, she was formally diagnosed with LAM. Because of the purported hormonal involvement of LAM at the time, the patient began treatment with long acting medroxyprogesterone (Depo-Provera) to limit the progression of LAM. Although the treatment induced chemical menopause as intended, it failed to prevent further progression of the disease. At age 29, she underwent a bilateral oophorectomy, but again this did not prevent further disease progression. Following continued decline in lung function, the patient underwent a bilateral lung transplant at 37 yr of age. At this point, the Wilms’ tumor histology was reviewed and recharacterized as a renal AML.

**Figure 1.**
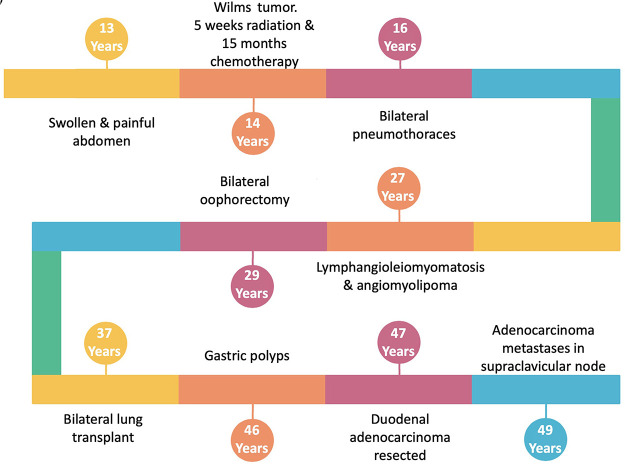
Overview time line of the patient's clinical history.

At age 46, gastroscopy enabled the identification and resection of a single inflammatory polyp and several fundic polyps. The following year, duodenal biopsies identified a tubular adenoma with focal high-grade dysplasia, as well as a hyperplastic polyp. In the same year, the patient underwent laparotomy. Both cholecystitis and cholelithiasis were noted, prompting cholecystectomy. Resection of sections D2, D3, and D4 of the duodenum and ∼50 cm of the jejunum was required to remove a mucinous duodenal adenocarcinoma and a second independent tumor separated by ∼33 mm of normal mucosa. Immunohistochemistry of the adenocarcinoma demonstrated loss of MSH2 and MSH6 proteins. Two years postresection of the duodenal adenocarcinoma, fine-needle aspirate of a supraclavicular lymph node identified metastatic cells consistent with the duodenal primary. An overview of the patient's clinic history is shown in Figure 1.

Initial genotyping in the patient (between 49 and 52 yr of age) was performed as per routine clinical care in a National Association of Testing Authorities/Royal College of Pathologists of Australasia (NATA/RCPA)-accredited laboratory. Sequencing interrogated the DNA mismatch repair genes *EPCAM*, *MLH1, MSH2, MSH6, PMS2, POLD1*, and *POLE*, with no pathogenic variants identified. As such, the relationship between LAM, AML, and the duodenal adenocarcinoma remained unknown. Therefore, we investigated the genetic and epigenetic relatedness of these malignancies with three objectives: First, we sought to investigate the presence of germline *TSC1* and *TSC2* mutations, to confirm whether both LAM and AML were sporadic or TSC-associated. Second, we investigated predicted driver mutations/epimutation in these malignancies to understand their pathogenesis. Finally, we used bioinformatic analyses to deconvolute subclonal composition of the duodenal adenocarcinoma.

We also sought to explore actionable driver mutations within subclone populations with hopes of identifying therapeutic targets in a precision oncology context. In doing so, we sought not only to deepen the understanding of the co-development of the malignancies in the patient, but also to provide evidence of the applicability in using subclonal deconvolution analyses to identify potentially actionable mutations in heterogenous tumor samples.

### Genomic Analyses

Whole-genome sequencing on peripheral blood was used to investigate the germline status of TSC-associated genes in the patient. Filtering of annotated genomic single-nucleotide variants (SNVs) using BCFtools identified four variants within the *TSC1*, *TSC2*, and *TBC1D7* genes. Two intronic variants of unknown significance were identified in *TSC1* and one in *TBC1D7*. In addition, a heterozygous single base substitution (C > A) was identified at a chromosomal location of 16:2129399 within *TSC2*, producing a premature stop codon (Table 1). Using American College of Medical Genetics and Genomics (ACGM) classifications, the variant is reported as “Pathogenic” (1c) with supporting evidence PM2, PVS1, and PP5. Heterozygosity for this variant was independently validated by the clinically accredited Canterbury Health Labs. Similarly, genomic variants in the DNA mismatch repair genes *EPCAM, MLH1*, *MSH2*, *MSH6*, *PMS2, POLD1*, and *POLE* were investigated. No coding variants were found in any of these genes.

**Table 1.** Truncating *TSC2* variant table

Gene	Chromosome	HGVS DNA reference	HGVS protein reference	Variant type	Predicted effect	Genotype
*TSC2*	16	NC_000016.9: g.212399C > T NM_000548.5 (TSC2): c.3254C > A	NP_000539.2 (TSC2): p.(Ser1085Ter)	Nonsense	Truncation	Heterozygous

### Investigating Epigenetic and Somatic Driver Mutations

**Figure 2.**
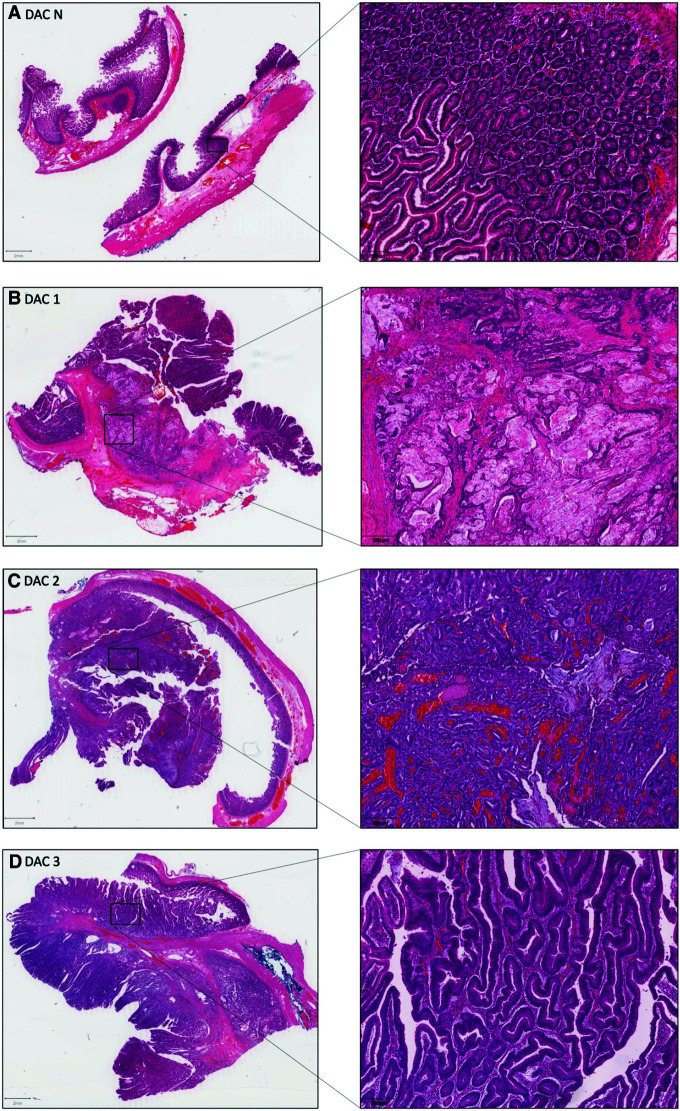

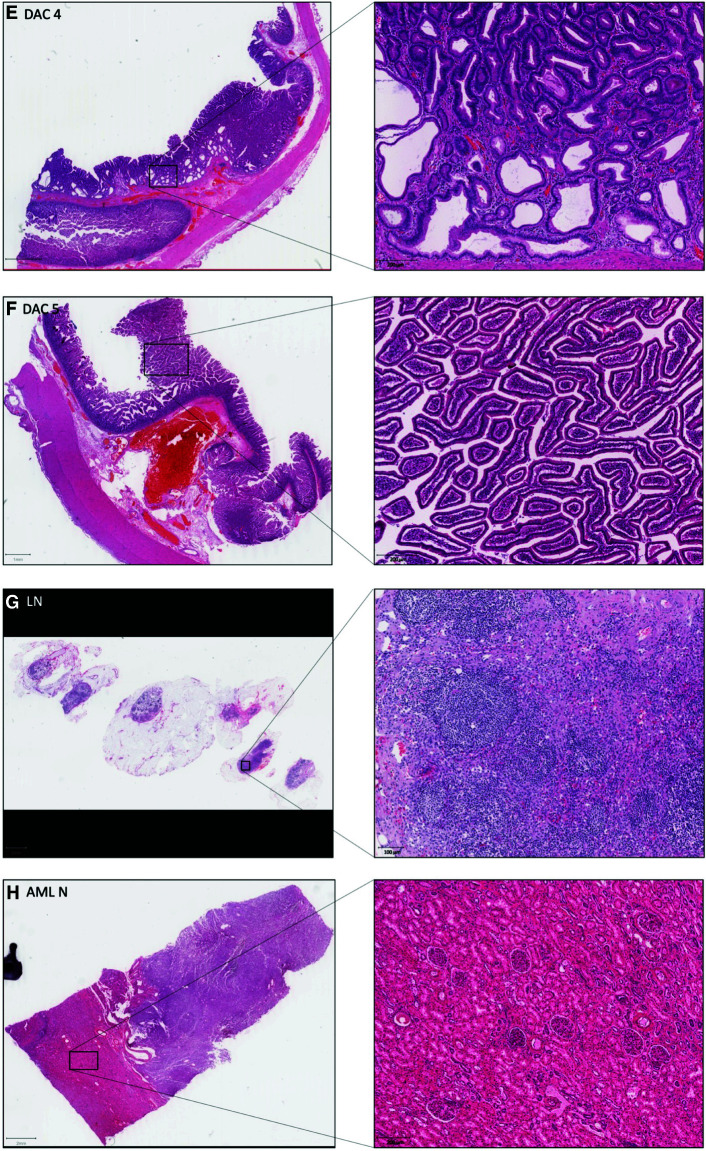

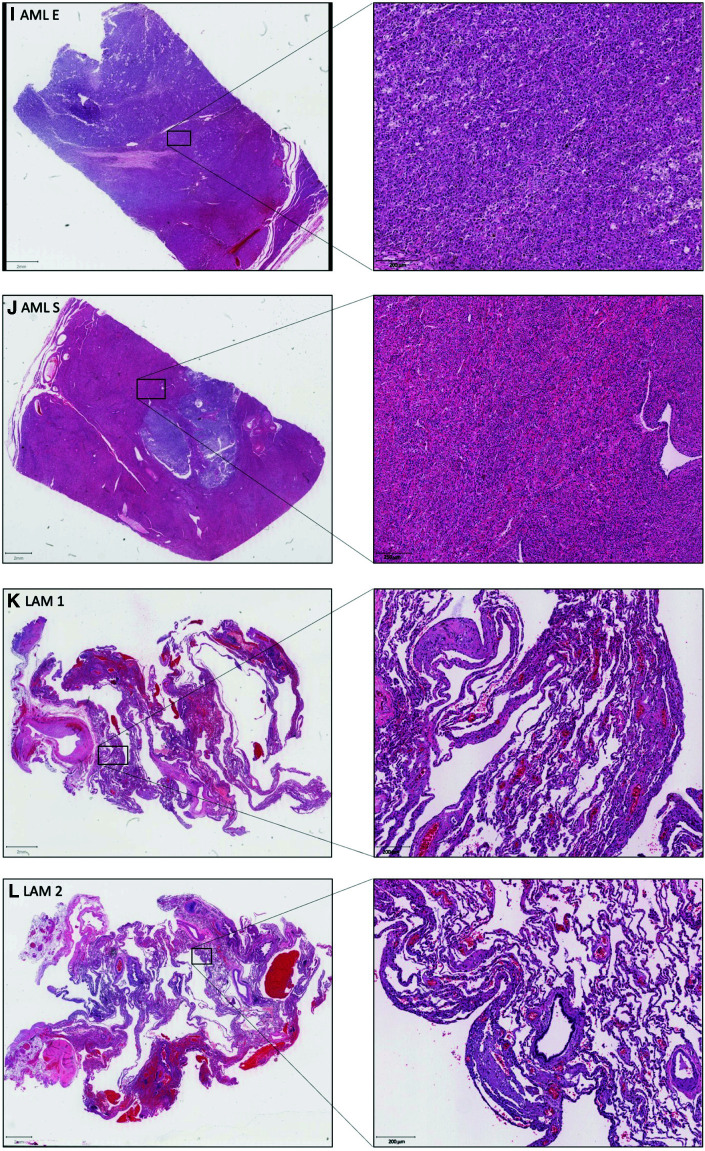
Hematoxylin and eosin stain of formalin-fixed paraffin-embedded tissue samples. (*A*) Adjacent normal duodenal mucosa tissue (DAC N). (*B*) Mucinous duodenal adenocarcinoma with infiltration into the duodenal wall/muscularis layers (DAC 1). (*C*) Adenomatous polyp exhibiting high-grade dysplasia and mucinous foci (DAC 2). (*D*) Sessile polyp exhibiting high-grade dysplasia (DAC 3). (*E*) Sessile polyp with low-grade dysplasia associated with second tumor (DAC 4). (*F*) Polyp with low-grade dysplasia (DAC 5). (*G*) Duodenal lymph nodes with smooth-muscle infiltrate (LN). (*H*) Adjacent normal kidney tissue with tubules and glomeruli visible (AML N). (*I*) Angiomyolipoma with epithelioid differentiation (AML E). (*J*) Angiomyolipoma with smooth-muscle differentiation (AML S). (*K*) Lung tissue with proliferative smooth-muscle nodule and association thickening of the alveolar septa consistent with lymphangioleiomyomatosis (LAM 1). (*L*) Progressive lymphangioleiomyomatosis lung pathology with normal alveoli, thickened alveolar septa, LAM cell nodules, and cystic spaces (LAM 2). (*Figure continues on following pages*.)

The Illumina TruSight Oncology (TSO) 500 Sequencing panel was used to interrogate the coding sequence of more than 500 cancer-associated genes in 12 formalin-fixed paraffin-embedded (FFPE) tissue samples from the patient (Fig. 2A–L). Binary base call (BCL) files generated from sequencing were processed as described in Illumina's TruSight Oncology 500 v2.0 Local App User Guide.

In total, 13,051 uncollapsed variants were called across the 12 FFPE tissue samples. CancerVar predicted a total of 25 independent pathogenic/likely pathogenic variants across the 12 tissue samples, whereas ClinVar predicted a total of 14 independent pathogenic variants. Twenty-nine independent somatic variants encoding premature stop codons and 49 independent frameshift variants were called. A list of predicted gene amplifications and deletions called can be found in Supplemental Table 1. No RNA splice variants or fusions were predicted.

Because of the identification of a pathogenic germline *TSC2* variant, somatic second hits were investigated in all AML and LAM samples. Interestingly, no evidence of somatic LOH was observed, with AML and LAM samples maintaining ∼64% (32/50) of reads as the reference allele at position 16:2129399 (Table 2). However, sequencing reads across these loci were inconsistent between samples (Supplemental Fig. S1). An additional frameshift deletion (CAG > C) mutation was observed at position 16:2134689 in ∼28% (11/39) of reads in the smooth-muscle-differentiated AML sample (Supplemental Fig. S2).

**Table 2.** Investigating somatic loss-of-heterozygosity *TSC2* mutations

Gene	Sample	HGVS DNA reference	HGVS protein reference	Variant type	Predicted effect	Alternate allelic fraction
*TSC2*	AML N	NC_000016.9: g.212399C > T NM_000548.5 (TSC2): c.3254C > A	NP_000539.2(TSC2_i001):p.(Ser1085Ter)	Nonsense	Truncation	0.31 (4/13)
*TSC2*	AML E	NC_000016.9: g.212399C > T NM_000548.5 (TSC2): c.3254C > A	NP_000539.2(TSC2_i001):p.(Ser1085Ter)	Nonsense	Truncation	0.4 (4/10)
*TSC2*	AML S	NC_000016.9: g.212399C > T NM_000548.5 (TSC2): c.3254C > A	NP_000539.2(TSC2_i001):p.(Ser1085Ter)	Nonsense	Truncation	0.35 (6/17)
*TSC2*	AML S	NC_000016.9:g.2134693_2134693del NM_000548.3(TSC2_v001):c.4470-4471del	NP_000539.2(TSC2_i001):p.(Lys1491Serfs*32)	Nonsense	Frameshift	0.28 (11/39)
*TSC2*	LAM 1	NC_000016.9: g.212399C > T NM_000548.5 (TSC2): c.3254C > A	NP_000539.2(TSC2_i001):p.(Ser1085Ter)	Nonsense	Truncation	0.5 (1/2)
*TSC2*	LAM 2	NC_000016.9: g.212399C > T NM_000548.5 (TSC2): c.3254C > A	NP_000539.2(TSC2_i001):p.(Ser1085Ter)	Nonsense	Truncation	0.38 (3/8)

**Figure 3.**
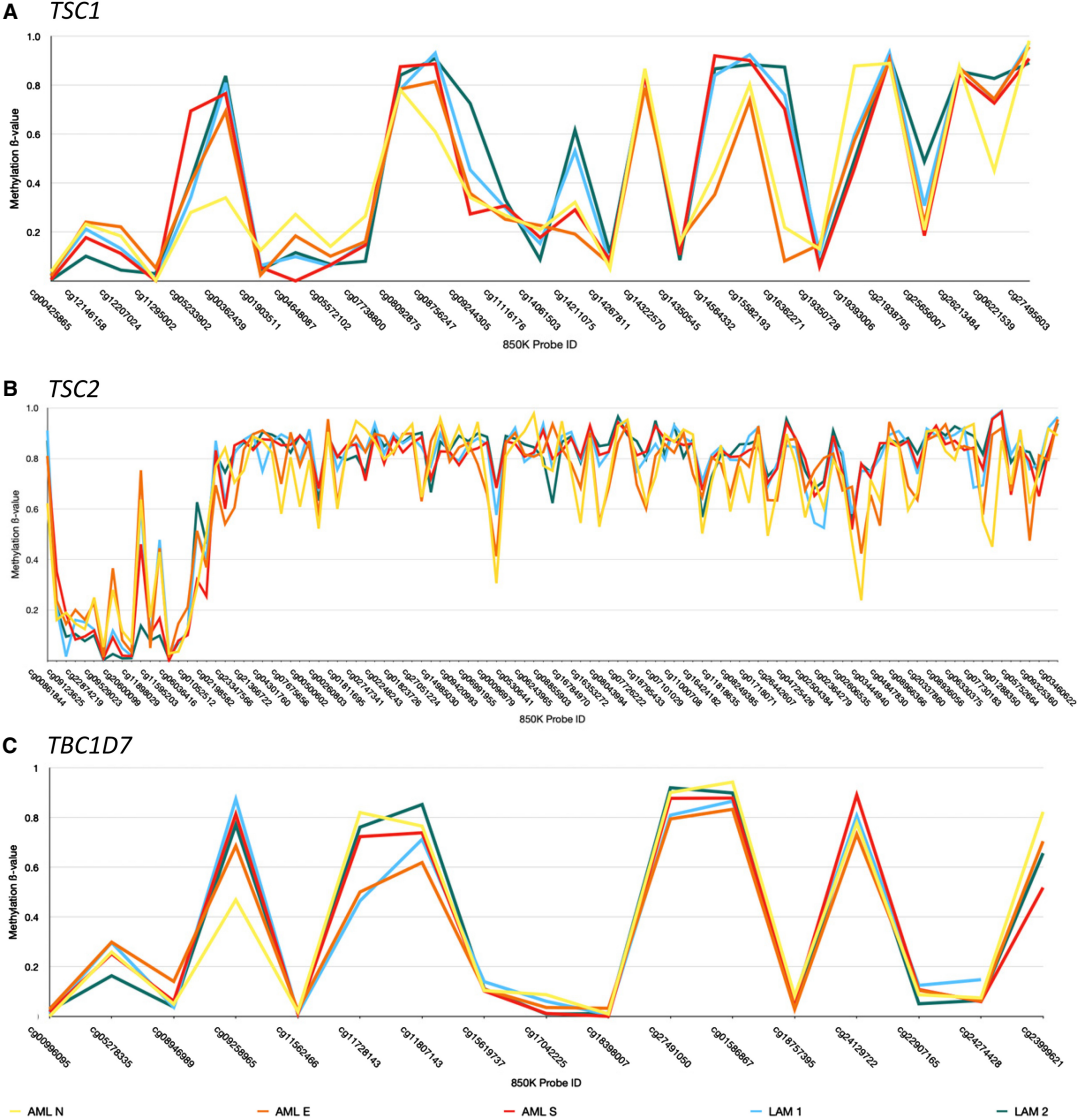
DNA methylation β values generated TSC-associated genes (*A*) *TSC1*, (*B*) *TSC2*, and (C) *TBC1D7*. The *x*-axis entails Illumina Infinium probe IDs. The *y*-axis shows the methylation β value.

In the absence of somatic *TSC2* LOH in both AML and LAM samples, DNA methylation–based epimutations in predicted TSC-driver genes (*TSC1*, *TSC2*, and *TBC1D7*) were investigated. No major deviations in DNA methylation β values were observed between samples in any TSC-associated gene (Fig. 3).

**Figure 4.**
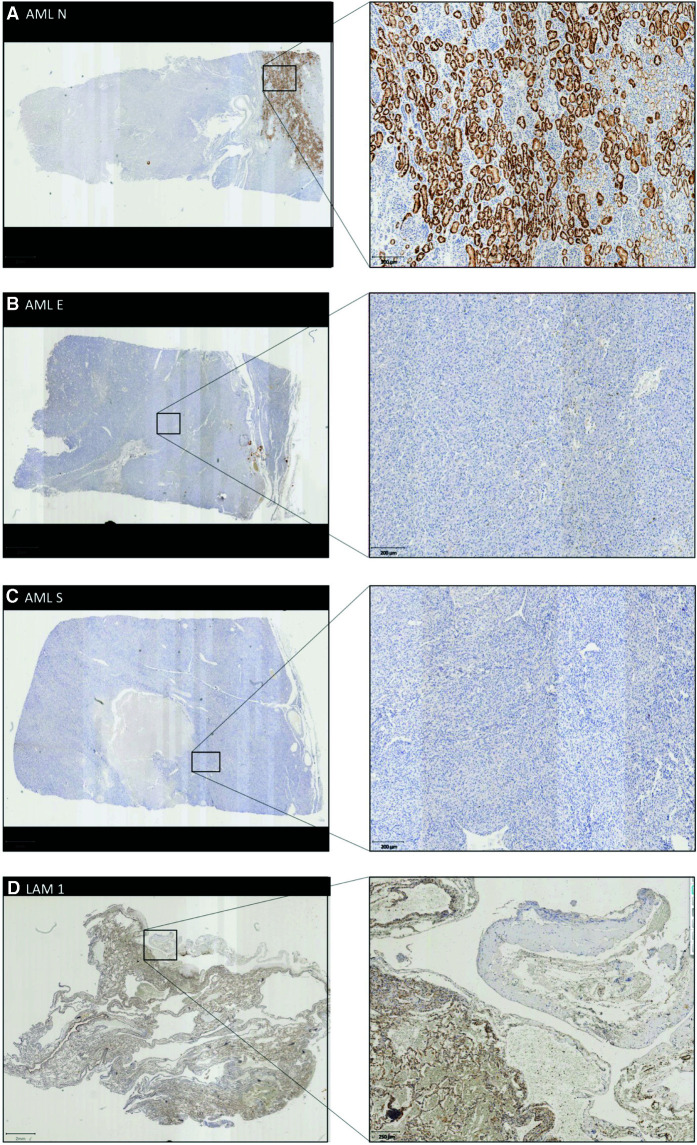
Immunohistochemistry demonstrating tuberin expression. (*A*) AML N—Adjacent normal kidney tissue with tuberin staining throughout tubules. (*B*) AML E—Epithelioid differentiated angiomyolipoma, no visible tuberin expression. (*C*) AML S—Smooth-muscle-differentiated angiomyolipoma, no visible tuberin expression. (*D*) LAM 1—Congested LAM lung tissue with visible tuberin staining in normal alveoli, but absent in proliferative smooth-muscle nodule (LAM). LAM 2—not shown, because of limited tissue samples.

Subsequently, immunohistochemistry was used to assess whether functional tuberin was expressed in AML and LAM samples. Of interest, the normal tissue adjacent to the angiomyolipoma displayed considerably variable tuberin expression, with light but diffuse cytoplasmic staining as well as apparent tuberin aggregates within some renal tubules (Fig. 4A). In contrast, both epithelioid- and smooth-muscle-differentiated angiomyolipoma displayed complete loss of tuberin expression (Fig. 4B,C). In the lung tissue, tuberin expression was limited to normal alveoli and thickened septa, but absent from LAM nodules (Fig. 4D).

Using immunohistochemistry, loss of MSH2 and MSH6 had been reported at the protein level in the duodenal adenocarcinoma in the pathologist's diagnostic report. As such, somatic mutations were investigated in the DNA mismatch repair genes *MLH1*, *MSH2*, *MSH6*, and *PMS2*.

In total, four independent somatic variants in *MLH1*, three variants in *MSH6*, and one variant *PMS2* were identified in the duodenal samples. No variants were called within *MSH2*. Consistent with previous genetic testing, all variants identified were predicted to be silent, producing no change in the final polypeptide sequence. Using annotated small somatic variants, tumor mutational burden (TMB) was calculated for each of the duodenal samples. TMB provides a measure of accumulated somatic mutations per megabase (Mb) of DNA. The postulated mismatch repair deficiency of the tumor is supported with a progressive increase in mutations/Mb DNA through the progressive polyps, polyps with high-grade dysplasia, and the adenocarcinoma (Table 3).

**Table 3.** Tumor mutational burden (TMB) in duodenal samples

	TMB sites	TMB (mutations/Mb)
DAC N—adjacent normal duodenum	0	0
DAC 5—polyp with low-grade dysplasia	0	0
DAC 4—sessile polyp with low-grade dysplasia	8	8.6
DAC 3—sessile polyp with high-grade dysplasia	7	53.2
DAC 2—adenomatous polyp with high-grade dysplasia	32	79.2
DAC 1—mucinous duodenal adenocarcinoma	79	82.7
LN—duodenal lymph nodes	1	3.1

**Figure 5.**
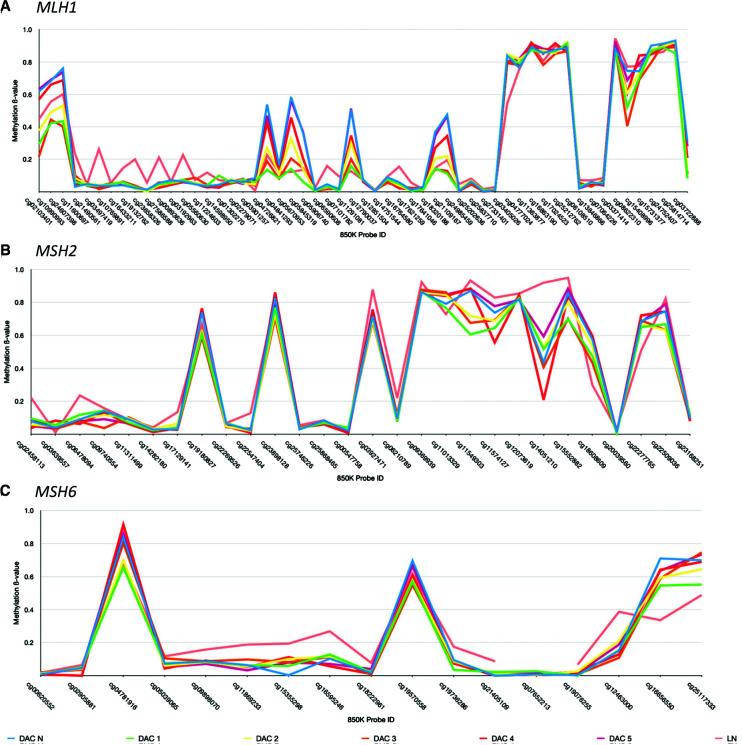
DNA methylation β values generated across the DNA mismatch repair genes (*A*) *MLH1*, (*B*) *MSH2*, and (*C*) *MSH6* genes. Data are not shown for PMS2 because of the low number of methylation probes. The *x*-axis entails Illumina Infinium probe ID. The *y*-axis demonstrates methylation β value.

Epigenetic silencing of the DNA mismatch repair genes *MLH1*, *MSH2*, *MSH6*, and *PMS2* via DNA hypermethylation was investigated in duodenum-associated samples. No major deviations in methylation β values were observed between tumor or adjacent normal tissue (Fig. 5). Interestingly, a trend of decreasing *MLH1* gene promotor methylation is observed in the progression of polyps with low- to high-grade dysplasia through to adenocarcinoma.

### Phylogenetic Analyses and Subclone Deconvolution in Duodenum-Associated Samples

**Figure 6.**
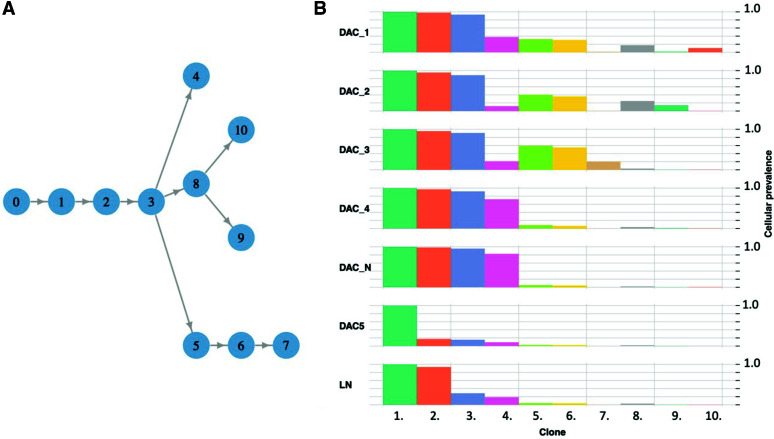
Subclone deconvolution analyses performed on duodenal samples using PhyloWGS. (*A*) Predicted clonal evolution within duodenal samples. (*B*) Predicted cellular prevalence of clones across duodenal samples.

As no predicted driver mutations were identified in the duodenal adenocarcinoma or dysplastic polyps displaying high- or low-grade dysplasia (Fig. 2B–F), deeper computational analyses were used using a subclonal composition reconstruction method, PhyloWGS (Deshwar et al. 2015). In total, 10 subclone populations were predicted within the six duodenal samples (Fig. 2A–F). Interestingly, subclone 8 had increased prevalence within the mucinous adenocarcinoma (Fig. 2B) and adenomatous polyp (Figs. 2C and 6). Moreover, this clone was predicted to contain the EGFR (Ala289Val) variant (Table 4). Supporting this, the Ala289Val variant was called in the mucinous adenocarcinoma and adenomatous polyp samples (Table 4). No reads of the EGFR A289V variant were found in other duodenal samples. Similarly, the CDH1 (Leu452Arg) variant was identified in mucinous adenocarcinoma and adenomatous polyp samples in ∼13% (11/87) and ∼19% (10/54) of reads for the locus, respectively (Table 4).

**Table 4.** Investigating somatic driver mutations in duodenal tissue samples

Gene	Sample	HGVS DNA reference	HGVS protein reference	Variant type	Predicted effect	Alternate allelic fraction
*ERCC2*	DAC 3	c.553C > T NC_000019.9:g.45868137G > A NM_000400.4(ERCC2_v001):c.553C > T	NP_000391.1(ERCC2_i001):p.(Arg185Trp)	Missense	Substitution	0.3 (6/20)
*ERCC2*	DAC 2	c.553C > T NC_000019.9:g.45868137G > A NM_000400.4(ERCC2_v001):c.553C > T	NP_000391.1(ERCC2_i001):p.(Arg185Trp)	Missense	Substitution	0.26 (7/27)
*ERCC2*	DAC 1	c.553C > T NC_000019.9:g.45868137G > A NM_000400.4(ERCC2_v001):c.553C > T	NP_000391.1(ERCC2_i001):p.(Arg185Trp)	Missense	Substitution	0.17 (6/36)
*DNMT3A*	DAC 3	NC_000002.11:g.25523072C > A NM_001320892.2(DNMT3A_v001):c.113G > T	NP_001307821.1(DNMT3A_i001):p.(Arg38Leu)	Missense	Substitution	0.41 (9/22)
*DNMT3A*	DAC 2	NC_000002.11:g.25523072C > A NM_001320892.2(DNMT3A_v001):c.113G > T	NP_001307821.1(DNMT3A_i001):p.(Arg38Leu)	Missense	Substitution	0.25 (8/32)
*DNMT3A*	DAC 1	NC_000002.11:g.25523072C > A NM_001320892.2(DNMT3A_v001):c.113G > T	NP_001307821.1(DNMT3A_i001):p.(Arg38Leu)	Missense	Substitution	0.26 (11/43)
*CDH1*	DAC 2	NC_000016.9:g.68849452T > G NM_004360.3(CDH1_v001):c.1355T > G	NP_004351.1(CDH1_i001):p.(Leu452Arg)	Missense	Substitution	0.13 (11/87)
*CDH1*	DAC 1	NC_000016.9:g.68849452T > G NM_004360.3(CDH1_v001):c.1355T > G	NP_004351.1(CDH1_i001):p.(Leu452Arg)		Substitution	0.19 (10/54)
*EGFR*	DAC 2	NC_000007.13:g.55221822C > T NM_201282.2(EGFR_v001):c.866C > T	p.A289V NP_598439.1(EGFR_i001):p.(Ala289Val)	Missense	Substitution	0.14 (10/72)
*EGFR*	DAC 1	NC_000007.13:g.55221822C > T NM_201282.2(EGFR_v001):c.866C > T	p.A289V NP_598439.1(EGFR_i001):p.(Ala289Val)		Substitution	0.15 (16/104)
*KIT*	LN	NC_000004.11:g.55599320G > A NM_000222.3(KIT_v001):c.2446G > A	NP_000213.1(KIT_i001):p.(Asp816Asn)	Missense	Substitution	0.08 (3/37)

Clones 5 and 6 were predicted to have highest cellular prevalence (Fig. 6B) in the mucinous adenocarcinoma, adenomatous polyp, and sessile polyp (Fig. 1D) samples. Likewise, mutations in ERCC2 (Arg185Trp) and DNMT3A (Arg38Leu) were identified across the three samples. Finally, the activating KIT (Asp816Asn) variant was predicted in clone 10 and limited to duodenum-associated lymph nodes. Importantly, none of the somatic ERCC2 (Arg185Trp), DNMT3A (Arg38Leu), or KIT (Asp816Asn) variants were identified within germline whole-genome sequencing data. These subclone deconvolution analyses suggest no single mutation was sufficient for tumorigenesis. Rather, the subsequent accumulation of multiple somatic mutations in multiple tumor-suppressor and proto-oncogenes enabled unrestricted cell growth and progression into malignancy.

## DISCUSSION

Hamartomatous presentations of the autosomal dominant disorder TSC typically develop following LOH mutations in the *TSC2* genes (Green et al. 1994). However, this assertion is becoming increasingly challenged for other manifestations of TSC (Jozwiak et al. 2008).

In this report, we describe a case study on a single TSC patient initially reported as sporadic LAM with a history of Wilms’ tumor. Although a germline premature truncating mutation was identified in one of the patient's *TSC2* alleles, no evidence of somatic LOH mutations or aberrations in DNA methylation were found in the LAM or AML samples. Although an additional frameshift deletion in *TSC2* was identified in smooth-muscle differentiation AML in ∼28% (11.39) of reads, it is unlikely to act as the second hit because of the apparent absence in the epithelioid differentiated AML sample. However, poor DNA quality in the AML samples (data not shown) may also contribute to low sequencing read depths at this locus in the epithelioid differentiated AML sample. Then again, variants of unknown significance may produce functional changes including the introduction of novel splice sites (Mayer et al. 2000). Moreover, intronic SNVs may act as a second hit for LOH by introducing splice sites as demonstrated in the Eker rat model (Kobayashi et al. 1997). As the TSO500 panel utilizes exome sequencing, it is possible that intragenic variants contributing to the pathogenesis of both LAM and AML tissues in this patient were not analyzed.

Meanwhile, immunohistochemical staining for tuberin demonstrated loss of the tumor suppressor protein in angiomyolipoma and proliferative LAM nodules. Of note, adjacent normal kidney tissue also displayed apparent aberrant tuberin distribution with concentrated foci within some renal tubules. Although an endogenous peroxidase blocking step was performed during the immunohistochemistry, it may have been of insufficient length. As such, the apparent intense tuberin staining in the renal tubules may result from persistent secreted peroxidases within the renal tubules (Whitin et al. 2002). Alternatively, these results may suggest that additional mechanisms could regulate tuberin at the protein level. One such mechanism could involve increased degradation by the proteasome following phosphorylation of protein kinase B (AKT) (Plas and Thompson 2003). Moreover, aberrations in tuberin mRNA are observed in other manifestations of TSC, suggesting tuberin expression may be altered at the transcriptional level (Kerfoot et al. 1996; Mizuguchi et al. 1997). Last, the selectivity of conventional tuberin antibodies against the tuberin splice isoforms is unknown. Additional research and analyses will be required to fully elucidate the mechanism behind the absent tuberin expression and subsequent pathogenesis of the TSC-associated malignancies in this patient.

In addition to multiple manifestations of TSC, the patient had multiple duodenal polyps as well as a mucinous duodenal adenocarcinoma. Although immunohistochemical analyses had demonstrated loss of the DNA mismatch repair genes *MSH2* and *MSH6*, no evidence of germline mutations, somatic mutations, nor somatic epimutations were observed. Rather, the transition to malignancy may have arisen following the accumulation of several successive mutations. First, the *ERCC2* (Arg185Trp) variant was identified in all duodenal samples exhibiting high-grade dysplasia. Although the functional significance of this variant has not been studied extensively, the introduction of tryptophan's with its structural indole ring at position 185 of the primary polypeptide sequence may produce steric hinderance, altering the protein's tertiary sequence. As such, this variant has been predicted to produce a highly deleterious tolerance index score (Fang et al. 2016). Subsequent to conformational changes in *ERCC2* protein structure, nucleotide excision repair mechanisms may become impaired, facilitating the accumulation of further mutations. This hypothesis is supported by the progressively increasing tumor mutation burden observed through the sessile polyp, adenomatous polyp, and duodenal adenocarcinoma samples. Subsequently, the acquisition of the invasive capabilities may have arisen with the *EGFR* (Ala289Val) variant, as observed in the adenocarcinoma and adenomatous polyp samples. This variant has been associated with increased invasiveness in glioblastoma models (Binder et al. 2018). Finally the *KIT* (Asp816Asn) variant was detected solely in the duodenal lymph node. This is consistent with other variants at this amino acid position increasing anchorage independent survival and proliferation (Green et al. 1994; Ning et al. 2001).

Of consideration, the lymphangiogenic growth factor VEGF-D is an important biomarker for LAM, with elevated serum VEGF-D correlated to increased disease severity (Young et al. 2010). As the levels of serum VEGF-D were not measured in this patient, whether this growth factor may have contributed to the metastasis of the duodenal adenocarcinoma remains unknown.

It must be acknowledged that the somatic sequencing performed using the TruSight Oncology 500 panel in these analyses were inconsistent, and in some cases had severely limited read depths (ranging from twofold to 47-fold coverage in AML samples, 40-fold to 57-fold in DAC, and sixfold coverage in LAM samples). We expect that these artifacts are related to the various ages and storage methods for these archival FFPE tissue samples, which may introduce limitations when interpreting these results.

Thus, this case represents an atypical presentation of a rare disease, with a lack of common features of TSC including neurological complications or hamartomatous manifestations of the skin. Moreover, the patient displays no discernible second hits driving the pathogenesis of the TSC-associated malignancies, AML and LAM. This warrants further investigations into the molecular features capable of enabling the progression from benign hamartomas into malignant PEComas. Finally, although limited reports of gastrointestinal manifestations of TSC are in the literature, we found no evidence of loss *TSC2* in contributing to the pathogenesis of the duodenal adenocarcinoma (Santos et al. 2015; Reis et al. 2020). Although there was also no evidence of loss of *TSC2* in the AML or LAM samples, the mucinous morphology of the duodenal adenocarcinoma and absence of additional PEComa markers suggest it is of an independent origin. Moreover, having undergone a bilateral lung transplant and thus requiring immunosuppressants produced a significant risk factor for malignancy in this patient.

In this single-patient case study, we cannot exclude the possibility that adjacent normal tissue used in comparisons may contain molecular aberrations, including somatic DNA mutations or epimutations, prior to the development of histopathological features. However, this is a general problem, which may be better addressed with deconvolution analyses. Thus, although intra-individual comparison of sequencing data provides an unparalleled means to identify accumulated somatic mutations, the applicability and usefulness of healthy control tissue cannot be overlooked.

The research performed throughout this case study highlights a significant challenge for the future of precision oncology. Indeed, whereas it is possible to generate vast amounts of data using various sequencing techniques, it is almost always bottlenecked at the interpretation step. That is, deciphering the consequences for the numerous variants of unknown significance requires substantial computational resources and time. Promisingly, new protocols and pipelines are being continuously developed to standardize this process. One such pipeline, PhyloWGS, shows utility, performing subclonal deconvolution in heterogeneous tumor samples. Although the complexity of the patient's case history limited treatment options, PhyloWGS called potentially actionable mutations including the *EGFR* (A289V) variant (Binder et al. 2018; Dai et al. 2018). Although this variant has also been called “Likely pathogenic” by CancerVar, it highlights the value of using multiple approaches during analysis. Furthermore, the subclonal deconvolution approaches not only demonstrate prevalence of a particular variant, but also insight into relative chronological origins. This requires further investigations in the context of phylogenetics and population dynamics analyses.

Thus, this case study both demonstrates the significant strides made in applying genomic sequencing technologies and highlights potential barriers capable of limiting progress in precision oncology.

## METHODS

### Investigating Germline Variants

#### DNA Extraction

Total DNA was extracted from 200 µL of peripheral blood from the patient using the QIAGEN Whole Blood Extraction kit.

#### Whole-Genome Sequencing

Whole-genome DNA sequencing was conducted by Macrogen Oceania using the Illumina Novaseq6000 sequencing platform. Preprocessing utilized a shotgun approach, with genomic DNA (gDNA) fragmented to ∼350 bp prior to use with the Illumina TruSeq DNA PCR Free library preparation kit protocol. DNA sequencing was performed for 150-bp paired end reads with a minimum of 30-fold coverage of the genome.

### Bioinformatic Pipeline for Analyzing Germline Mutations

#### Alignment and Simple Sample Calling Pipeline

Paired fastq files generated from the sequencing protocol were aligned to the human reference genome GRCh37 using BWA (version 0.7.17). Duplicated reads were identified using the MarkDuplicates function within the Picard tool (version 2.18.11). The GATK (version 3.8.2) tool BSQR (base quality score recalibration) was used to recalibrate the base quality scores. Average depth of sequencing coverage for each chromosome was calculated using the GATK tool DepthofCoverage. Comparison of read depth between autosomes and sex chromosomes enabled chromosome-based sex prediction. To call variants between the patient's whole-genome sequencing sample and GRCh37, the GATK HaploTypeCaller tool was used to produce a single sample genomic VCF.

#### Joint Genotyping and Refine Pipeline

Sequencing data processing was consistent with industry standard GATK best practice guidelines. The genomic VCF file generated in the alignment and simple sample calling pipeline was imported into a VCF database using the GATK (version 4.1.4.1) using the GenomicsDBImport tool. Joint genotyping was performed using the GATK tool GenotypeGVCFs. Variant quality score recalibration was undertaken separately for both single-nucleotide polymorphisms (SNPs) and indels using the GATK tool VariantRecalibrator.

#### Annotation and Filtering

Annotation and determination of functional significance for all variants was performed using Ensembl's variant effect predictor (VEP 98.3). Further annotations were performed using BCFtools, using data from gnomAD (Version r2.1). BCFtools was used to filter for variants based on gene symbols (*TSC1, TSC2*, and *TBC1D7*) and gnomAD allele frequency thresholds.

### Investigating Somatic Mutations in Fixed Tumor Samples

Next-generation sequencing was used on 12 FFPE tumors or associated normal tissue samples collected throughout the patient's history. Hematoxylin and eosin staining and immunohistochemistry of tissue samples was performed using the Leica Bond-RX automated staining system. Tuberin antibodies (Cell Signaling Technology 4308) were used at 1:400 dilutions. For each sample, six 10-µM sections were mounted on slides with areas of interest demarcated. Slide scraping, total nucleic acid isolation, and library preparation was performed by Grafton Clinical Genomics (University of Auckland).

#### Illumina TruSight Oncology 500 Sequencing Panel

To investigate the total coding regions of more than 500 cancer-associated genes, the Illumina TruSight Oncology 500 (TSO500) panel was used in collaboration with Grafton Clinical Genomics. The panel assesses both DNA and RNA from a single tissue sample, enabling assessment of SNVs, insertions, deletions, amplifications, splice variants, and gene fusions. Moreover, as the panel covers >1.9 Mb of DNA and numerous microsatellite regions, it may be used effectively to calculate both TMB and microsatellite instability (MSI), important biomarkers for patient responsiveness to immune checkpoint inhibitors. No modifications were made to the protocol available via the Illumina TSO500 Reference Guide, utilizing the Illumina NextSeq500 platform.

#### TruSight Oncology 500 Data Analyses

The data output from the TSO500 panel was analyzed in collaboration with Grafton Clinical Genomics using the TruSight Oncology 500 Local App (v2.0). Reference manuals for the TruSight Oncology 500 Local App are available at the following:

https://support.illumina.com/downloads/trusight-oncology-500-local-app-user-guide-1000000067616.html
https://support.illumina.com/content/dam/illumina-support/documents/documentation/software_documentation/trusight/trusight-oncology-500/trusight-oncology-500-local-app-v2-user-guide-1000000095997-02.pdf

### Investigating Genome-Wide DNA Methylation Profiles in Fixed Tumor Samples

#### Extraction of Nucleic Acids

For each of the 12 FFPE tissue samples, four 10-µM tissue microtome sections were collected onto glass microscopy slides. Excess paraffin wax was removed using a sterile razor, before collecting the remaining embedded tissue samples into 1.5-mL plastic tubes. Remaining paraffin within samples was removed using 400 µL of QIAGEN Deparaffinization Solution before proceeding to the QIAamp DNA FFPE Tissue Kit for DNA extraction. No modifications were made to the protocol.

#### Bisulfite Conversion

DNA extracted from FFPE samples was bisulfite-converted using the ZymoResearch EZ-96 DNA Methylation Kit.

#### Illumina FFPE Restoration

Because of the nature of DNA derived from FFPE tissue samples being highly fragmented, the Illumina Infinium HD FFPE DNA Restore kit was used to increase the integrity of the DNA prior to analyses via methylation arrays.

#### Illumina Infinium DNA Methylation Array

To investigate the methylation status of more than 850,000 CpG sites within the 12 bisulfite-converted and “restored” FFPE DNA samples, the Illumina Infinium HD MethylationEPIC (850K) array was used in collaboration with AgResearch (Invermay, Dunedin).

#### Clonal Deconvolution and Phylogenetic Reconstruction

Understanding the clonal evolution of individual tumors may help identify actionable driver mutations and thus provide insights into treatment responsiveness. To perform phylogenetic reconstruction from heterogenous tumor sequencing data, subclones must be identified using variant allele frequencies before their evolutionary history can be reconstructed. To perform subclonal deconvolution we have used PhyloWGS, which provides increased accuracy by predicting subclonal populations and reconstructing their evolutionary history in parallel. PhyloWGS was run on 1462 SNVs called by the TruSight Oncology 500 panel. The software was run with default parameter settings and four Markov chain Monte Carlo (MCMC) chains. The most likely tree was retained.

## ADDITIONAL INFORMATION

### Data Deposition and Access

The variants were submitted to ClinVar (https://www.ncbi.nlm.nih.gov/clinvar/) and can be found under accession number SCV002098935. Because of the identifiable nature of this rare disease, data from this project may be available on request from the corresponding author. Quality control metrics from somatic sequencing are available on request.

### Ethics Statement

Written informed consent was obtained from the patient for this case study. The University of Otago Human Ethics Committee (Health) has approved this research (H19/083).

### Acknowledgments

The authors thank the patient and her family for their willingness to enable this research project and their continued enthusiasm. The authors thank The New Zealand LAM Charitable Trust for facilitating this project since its conception. The authors thank Professor Stephen Robertson for his advice and guidance in navigating clinical genomics.

### Funding

Funding for this project was provided by The New Zealand Charitable Trust, The Maurice and Phyllis Paykel Trust, and the Cancer Research Trust. A.G. acknowledges support from the Royal Society Te Apa¯rangi through a Rutherford Discovery Fellowship (RDF-UOO1702). A.G. and J.C.M. were partially supported by the Ministry of Business, Innovation, and Employment of New Zealand through an Endeavor Smart Ideas grant (UOOX1912) and a Data Science Programmes grant (UOAX1932).

### Competing Interest Statement

The authors have declared no competing interest.

## Supplementary Material

Supplemental Material
