# Supplementary material for: Tuberous sclerosis complex: a complex case
Source: Cold Spring Harb Mol Case Stud. 2022 Apr;8(3):a006182. doi: 10.1101/mcs.a006182 (PMC9059781; doi:10.1101/mcs.a006182)
Supplement: Supplemental Material [file supp_8_3_a006182__DC1.html]

Supplemental Material 

# Tuberous sclerosis complex: a complex case

## Supplemental Material

- Supplemental\_Material.docx
