# Supplementary material for: Tuberous sclerosis complex: a complex case
Source: Cold Spring Harb Mol Case Stud. 2022 Apr;8(3):a006182. doi: 10.1101/mcs.a006182 (PMC9059781; doi:10.1101/mcs.a006182)

# Supplementary Tables:

Table S1: Predicted gene amplifications, deletions, RNA splice variants and fusions

|  | **Gene Deletion** | **Gene Amplification** |
| --- | --- | --- |
| **DAC 3** |  | - TFRC (CNA ~3.07 @ 100% tumor cellularity) |
| **DAC 4** |  | - ERBB2 (CAN ~3.01 @ 100% tumor cellularity) |
| **DAC 5** | - PDGFRA (CNA ~1.14 @ 100% tumor cellularity). - FGF5 (CNA ~1.12 @ 100% tumor cellularity). - FGF10 (CNA ~1.01 @ 100% tumor cellularity). - ESR1 (CNA ~1.03 @ 100% tumor cellularity). - RET (CNA ~1.25@ % tumor cellularity). - FGF6 (CNA ~1.33@ 100% tumor cellularity). - FGF14 (CNA ~0.97@ 100% tumor cellularity). - FGF7 (CNA ~1.18@ 100% tumor cellularity). - AR (CNA ~0.73@ 100% tumor cellularity). | - ERBB3 (CNA ~3.65 @ 100% tumor cellularity). - CDK4 (CNA ~2.91 @ 100% tumor cellularity). - ERBB2 (CAN ~3.07 @ 100% tumor cellularity). - ERCC1 (CNA ~3.27 @ 100% tumor cellularity). |
| **LN** | - NRG1 (CNA ~1.15@ 100% tumor cellularity). AR (CNA ~1.21@100 % tumor cellularity). | - MYC (CNA ~3.22@100 % tumor cellularity). - PTEN (CNA ~2.98@100 % tumor cellularity). - CDK4 (CNA ~3.38@100 % tumor cellularity). - ERCC1 (CNA ~3.47@100 % tumor cellularity). |

# Supplementary Figures:

Figure S1: Visualization of sequencing reads containing the reported *TSC2* truncating mutation (NP_000539.2(TSC2_i001):p.(Ser1085Ter)) in LAM and AML samples using Integrated Genome Browser (IGV).


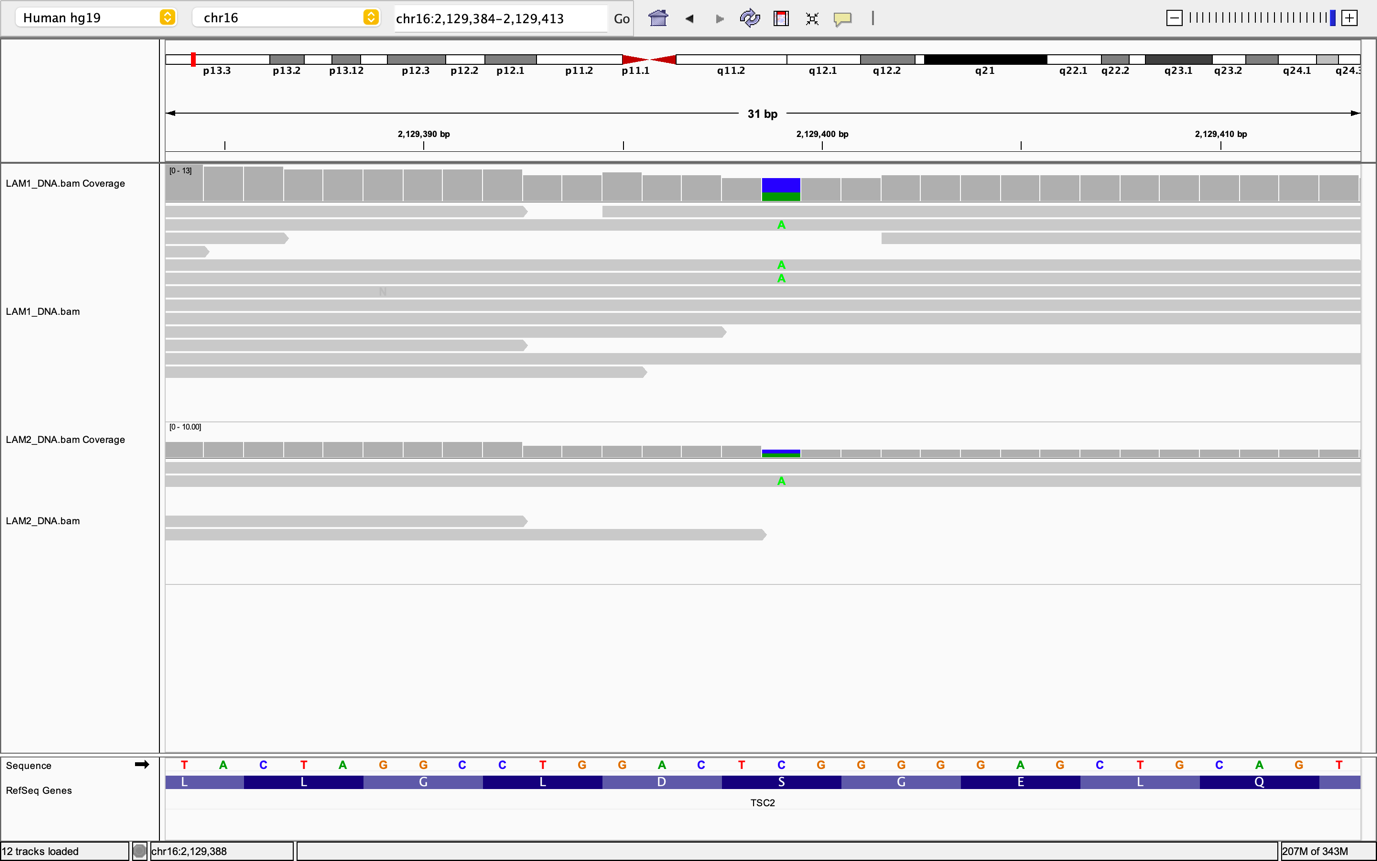


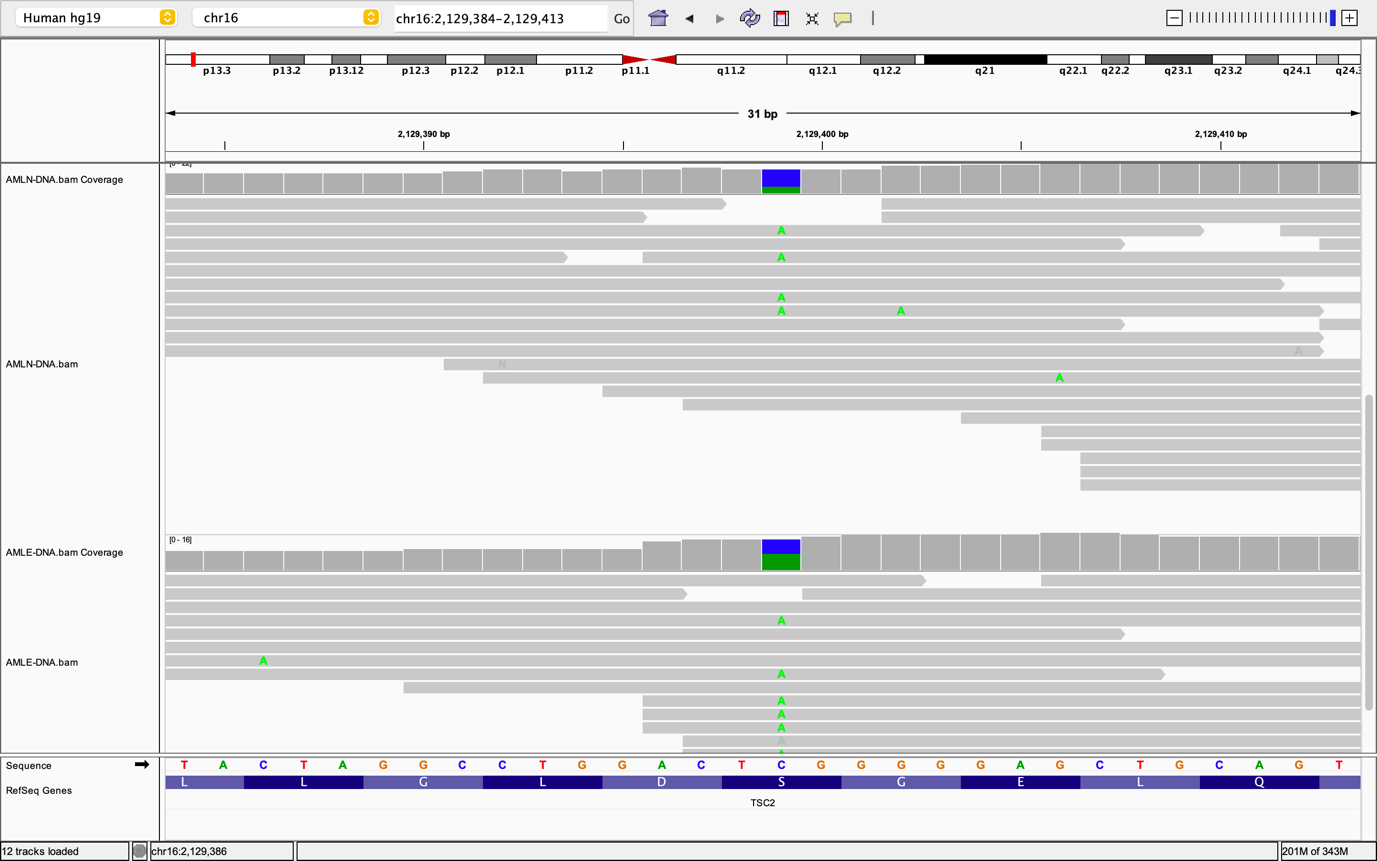


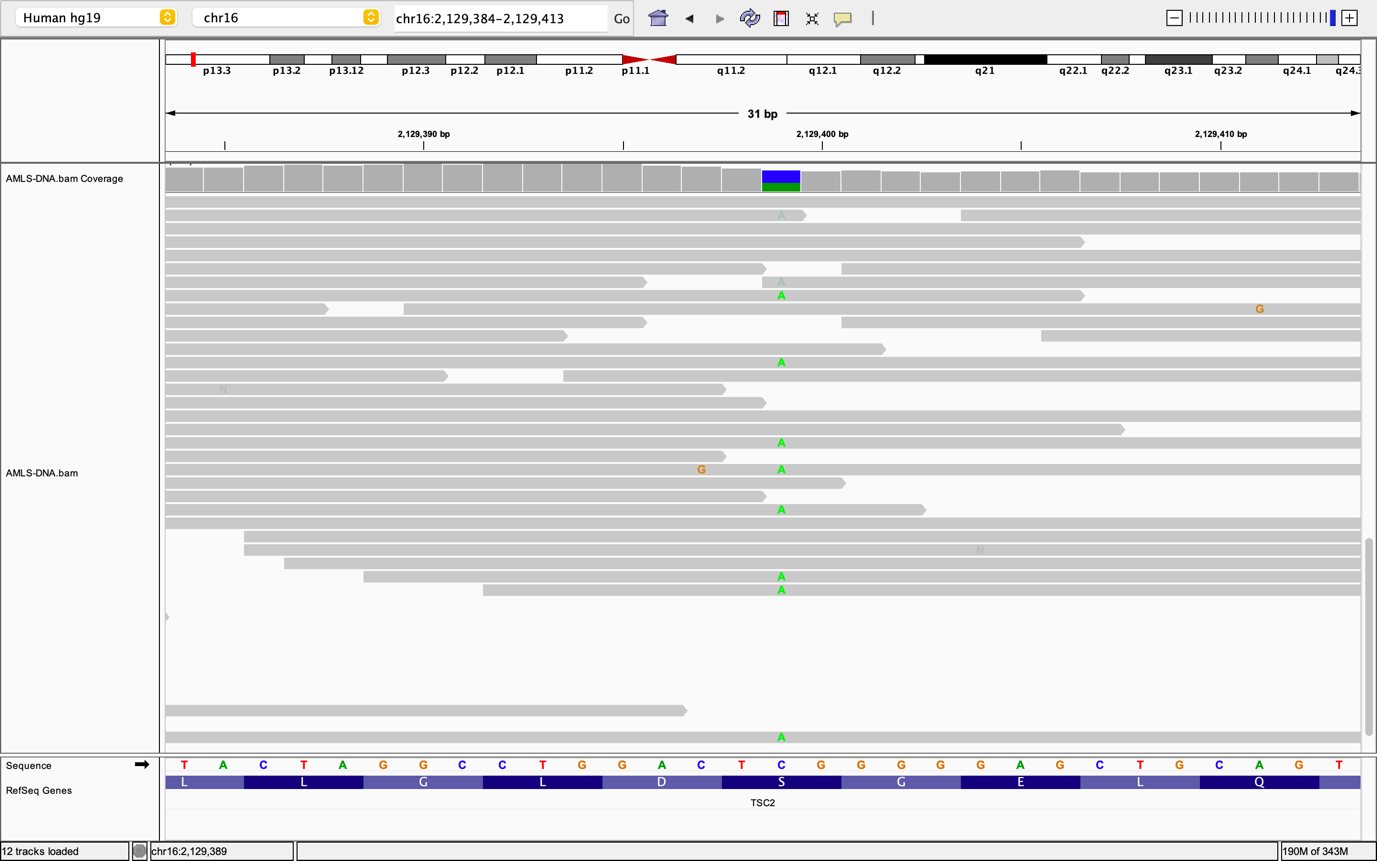


Figure S2: Visualization of sequencing reads containing the reported *TSC2* frameshift mutation (NP_000539.2(TSC2_i001):p.(Lys1491Serfs*32)) in the AML-S sample using Integrated Genome Browser (IGV).


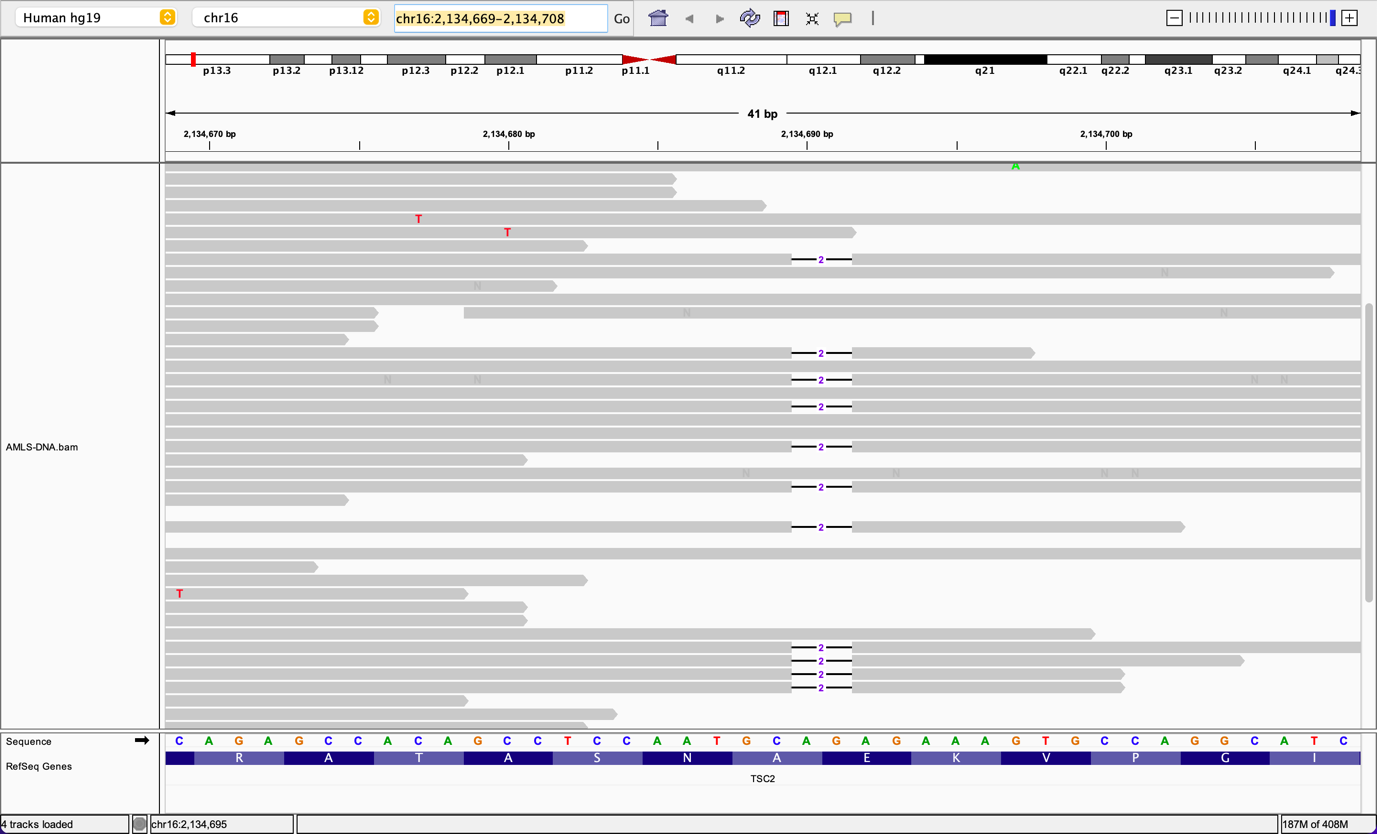

Supplement: Supplemental Material [file supp_mcs.a006182_Supplemental_Material.docx]
